# Lumen Expansion Facilitates Epiblast-Primitive Endoderm Fate Specification during Mouse Blastocyst Formation

**DOI:** 10.1016/j.devcel.2019.10.011

**Published:** 2019-12-16

**Authors:** Allyson Quinn Ryan, Chii Jou Chan, François Graner, Takashi Hiiragi

**Affiliations:** 1 Developmental Biology Unit, European Molecular Biology Laboratory (EMBL), 69117 Heidelberg, Germany; 2 Laboratoire Matière et Systèmes Complexes, Université Denis Diderot, Paris 7, CNRS UMR 7057, Condorcet Building 10 rue Alice Domon et Léonie Duquet, 75205 Paris Cedex 13, France; 3 Institute for the Advanced Study of Human Biology (WPI-ASHBi), Kyoto University, Kyoto, Japan

**Keywords:** early mammalian development, cell fate specification, lumenogenesis, cell sorting, self-organization, FGF signaling, mouse blastocyst, primitive endoderm, epiblast, vesicle release

## Abstract

Epithelial tissues typically form lumina. In mammalian blastocysts, in which the first embryonic lumen forms, many studies have investigated how the cell lineages are specified through genetics and signaling, whereas potential roles of the fluid lumen have yet to be investigated. We discover that in mouse pre-implantation embryos at the onset of lumen formation, cytoplasmic vesicles are secreted into intercellular space. The segregation of epiblast and primitive endoderm directly follows lumen coalescence. Notably, pharmacological and biophysical perturbation of lumen expansion impairs the specification and spatial segregation of primitive endoderm cells within the blastocyst. Luminal deposition of FGF4 expedites fate specification and partially rescues the reduced specification in blastocysts with smaller cavities. Combined, our results suggest that blastocyst lumen expansion plays a critical role in guiding cell fate specification and positioning, possibly mediated by luminally deposited FGF4. Lumen expansion may provide a general mechanism for tissue pattern formation.

## Highlights

• Lumenogenesis coincides with cytoplasmic vesicle release into intercellular space
• Mouse blastocyst epiblast-primitive endoderm segregation follows lumen expansion
• Reduced lumen expansion impairs cell fate specification and segregation
• Luminally deposited FGF4 expedites epiblast-primitive endoderm specification

## Graphical Abstract

**Figure:**
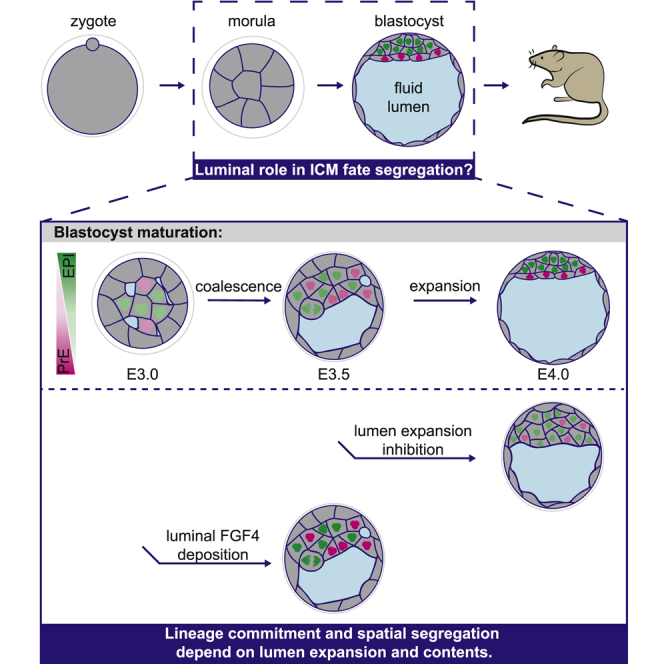

## Teaser

Mammalian pre-implantation development generates a blastocyst containing three spatially segregated cell lineages and a fluid lumen. Ryan et al. find that the specification and spatial segregation of epiblast and primitive endoderm lineages within the blastocyst are facilitated by lumen expansion and luminal FGF4 signaling.

## Introduction

Pre-implantation mouse development culminates with the formation of a blastocyst containing three spatially segregated cell lineages and an abembryonically localized fluid lumen. The molecular specification of the three cell lineages—trophectoderm (TE), epiblast (EPI), and primitive endoderm (PrE) —occurs sequentially (Artus et al., 2011, Chazaud et al., 2006, Rossant and Tam, 2009). Asymmetric divisions and differential contractility result in a 16-cell stage embryo containing apolar inner cells that upregulate inner cell mass (ICM) markers and polar outer cells that upregulate TE markers (Anani et al., 2014, Johnson and Ziomek, 1981, Korotkevich et al., 2017, Maître et al., 2016, Nishioka et al., 2009, Strumpf et al., 2005). Subsequent cleavage rounds to 32, 64, and 128-cell stages see the establishment of the EPI and PrE cell lineages. During the 32-cell stage, inner cells are stochastically biased toward either EPI or PrE identity on a molecular level (Chazaud et al., 2006, Guo et al., 2010, Ohnishi et al., 2014, Schrode et al., 2014). These biases are reinforced or changed during the following blastocyst development depending on cell position within the ICM, such that the EPI cells are surrounded by TE and PrE cells, which align along the luminal surface (Frankenberg et al., 2011, Plusa et al., 2008, Saiz et al., 2013). Fibroblast growth factor (FGF) signaling plays a key role in establishing the molecular identity of ICM cells (Chazaud et al., 2006, Kang et al., 2013, Krawchuk et al., 2013, Ohnishi et al., 2014, Yamanaka et al., 2010) and signals to both ICM lineages (Kang et al., 2017, Molotkov et al., 2017) as well as the trophectoderm (Goldin and Papaioannou, 2003).

**Figure 1:**
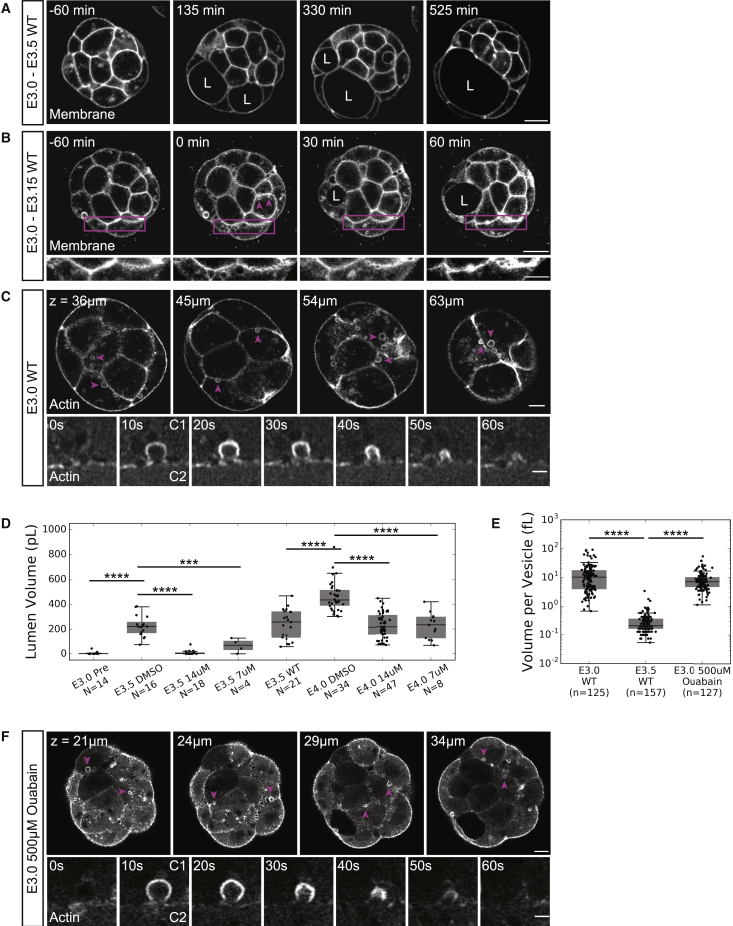
Blastocyst Cavities Are Partially Derived from Cytoplasmic Vesicles (A) Time-lapse of a representative embryo expressing a membrane marker undergoing lumen formation (L marks a lumen). *t* = 0 min when fluid accumulation is first detectable by automatic segmentation. Scale bar, 20 μm. (B) Time-lapse of the 1st h of fluid accumulation in an embryo expressing a membrane marker (L marks a lumen). *t* = 0 min when “string of pearls” microluminal structures are observed. Top row is full embryo view (magenta arrowheads highlight cytoplasmic vesicles, scale bar, 20 μm). Bottom row is insets of cell-cell interfaces indicated by magenta boxes in top row highlighting the appearance of “string of pearls”-like microlumina emergence and resolution (scale bar, 10 μm). (C) Z slices of phalloidin staining showing cortically-localized vesicles in an E3.0 embryo (top, magenta arrowheads highlight individual vesicles, scale bar, 10 μm). Time-lapse of vesicle secretion into intercellular space in a Lifeact-GFP E3.0 embryo (bottom, “C1” marks secreting cell, “C2” marks adjacent cell, scale bar, 2 μm). (D) Boxplot of volume for lumina in brefeldin A pharmacologically inhibited embryos. (E) Boxplot of volume for individual vesicles in WT embryos at E3.0 and E3.5, and ATP1 inhibited embryos at E3.0. (F) Z slices of an E3.0 embryo expressing Lifeact-GFP showing vesicle localization under Atp1 inhibition conditions (top, magenta arrowheads highlight individual vesicles, scale bar, 10 μm). Time-lapse of vesicle secretion into intercellular space under Atp1 inhibition conditions (bottom, “C1” marks secreting cell, “C2” marks adjacent cell, scale bar, 2 μm). ^∗∗∗^p < 0.001 ^∗∗∗∗^p < 0.0001. N = number of embryos. n = number of vesicles. For boxplots: central mark indicates the median; lower edge, 25%; upper edge, 75%; lower whisker, Q1 − (1.5 × IQR), where IQR = Q3−Q1; upper whisker, Q3 + (1.5 × IQR). See also Figure S1; Videos S1, S2, S3, and S4.

Concurrently with the specification of EPI and PrE, the blastocyst lumen begins to form and expand (Rossant and Tam, 2009). The major driver of fluid accumulation within the embryo is thought to be Atp1, a Na^+^/K^+^-ATPase, which forms an osmotic gradient across TE cells through its polarized basolateral expression (Wiley, 1984, Watson, 1992, Watson and Barcroft, 2001). Early phases of fluid accumulation result in multiple fluid pockets that preferentially localize at the base of TE cells before coalescing to form the abembryonic pole of the embryonic-abembryonic axis (Figure 1A; Dumortier et al., 2019, Motosugi et al., 2005). The apical domain of TE cells continues to face the external environment of the embryo throughout lumen expansion, which is inverted in comparison to typical cystic and tubular lumina in which the apical domain faces the lumen (Bryant and Mostov, 2008). Inner cells exposed to the lumen, PrE cells, do not undergo polarization and epithelialization until the final stages of pre-implantation development (E3.75–E4.0; Gerbe et al., 2008). Tight regulation and maintenance of the established apico-basal polarity and tight junction networks within the TE cells are required for blastocyst lumen formation and expansion (Eckert et al., 2004, Madan et al., 2007, Moriwaki et al., 2007).

Thus far, the function(s) of the blastocyst lumen are largely unknown. Depending on the tissue and developmental time, fluid lumina have widely varied functions including cell shape change (Gebala et al., 2016), nutrient delivery and absorption (Sobajima et al., 2014), left-right symmetry breaking (Essner et al., 2005), and even signaling niche establishment (Durdu et al., 2014). Interestingly, it has been shown in the mouse pancreas that failed tubulogenesis causes altered fate allocation ratios of progenitor cells due to changes in microenvironments (Kesavan et al., 2009). Despite the temporal correlation of EPI-PrE specification and lumen formation, the potential functions of the lumen to regulate fate specification and cell positioning have yet to be investigated. In this work, we examined early phases of blastocyst lumen formation and expansion in relation to cell fate specification and spatial position with an aim to better understand the interplay between cell fate specification and blastocyst morphogenesis.

## Results

### Widespread Secretion of Cytoplasmic Vesicles into Intercellular Space Drives Early Fluid Accumulation

Given the multipoint origin of the blastocyst lumen (Motosugi et al., 2005), we examined the first moments of extracellular fluid accumulation at high spatial resolution over multiple timescales (Figures 1A–1C). In addition to the previously reported multiluminal stage, we observed the unbiased appearance of microlumina arranged in a “string of pearls” like morphology at cell-cell interfaces throughout the embryo that progressively undergo coalescence over 2–3 h (Figure 1B). In embryos at a stage just prior to measurable separation of cell membranes due to fluid accumulation (approximately 84 h post-hCG (human chorionic gonadotropin), E3.0), we observed large, cortically-localized, actin-coated vesicles along the basolateral membranes of outer cells and ubiquitously along the membranes of apolar, inner cells (Figure 1C, top panel; Video S1). These vesicles are actively secreted into intercellular space in approximately 60 s (Figure 1C, bottom panel; Video S2). The presence and dynamic behavior of these vesicles persists through the early phases of luminal coalescence and expansion (E3.0–E3.25); however, such vesicles are no longer observable in embryos in which the lumen has expanded to occupy at least 50% of the total embryo volume (approximately 96 h post-hCG, E3.5; Figures 1E and S1A). Actin-coated vesicles in E3.5 embryos are significantly smaller than those in E3.0 embryos; they form cytoplasmic clusters, and no observable secretion events occur at the same time scale as that of E3.0 vesicles (Figures 1E and S1).

Video S1. Vesicles Localize to All Basolateral and Apolar Membranes Prior to Visible Extracellular Fluid Accumulation, Related to Figure 1 Z stack of actin signal (phalloidin) in a WT E3.0 embryo. Scale bar, 10 μm.

Video S2. Vesicles Are Actively Secreted into Intercellular Space, Related to Figure 1 Maximum intensity Z-projection time-lapse (time step = 10 s) of actin localization (Lifeact-GFP) during vesicle secretion in a WT E3.0 embryo. Scale bar, 5 μm.

To discern if vesicle release makes a measurable contribution to total luminal volume, embryos from early (E3.0–E3.5) and late (E3.5–E4.0) stages of lumen expansion were incubated in media containing Brefeldin A, a well-known inhibitor of COPII machinery (Miller et al., 1992, Helms and Rothman, 1992) that does not affect cell divisions (Figure S1B) or increase the frequency of apoptotic events (Figure S1C). Titrations of Brefeldin A revealed that its inhibitory effects on luminal volume can be modulated during early lumen expansion phases (Figure 1D) in agreement with the observation of vesicle release occurring primarily during early expansion (Figures 1C and S1A). To determine if vesicle release is linked to the Atp1 driven mechanism of fluid accumulation, we incubated embryos with ouabain, a well-known inhibitor of Atp1 activity (Bagnat et al., 2007, Manejwala et al., 1989), for 2 h prior to fluid accumulation onset. We observed that the vesicle release mechanism is still present in embryos cultured with ouabain (Figure 1F, top panel; Figure 1E; Video S3). The time it takes a single vesicle in Atp1 inhibited embryos to be secreted appears similar to that of WT embryos (Figure 1F, bottom panel; Video S4). These results suggest that vesicle secretion as a fluid accumulation mechanism is independent of Atp1.

Video S3. WT Localization of Vesicles Is Maintained in Atp1 Inhibited Embryos, Related to Figure 1 Z stack of actin signal (Lifeact-GFP) in an Atp1 inhibited E3.0 embryo. Scale bar, 10 μm.

Video S4. Vesicles Continue to Be Secreted into Intercellular Space during Atp1 Inhibition, Related to Figure 1 Maximum intensity Z-projection time-lapse (time step = 10 s) of actin localization (Lifeact-GFP) during vesicle secretion in an Atp1 inhibited E3.0 embryo. Scale bar, 5 μm.

### Early Luminal Structures Are Marked with Apical Proteins and Contain FGF4

**Figure 2:**
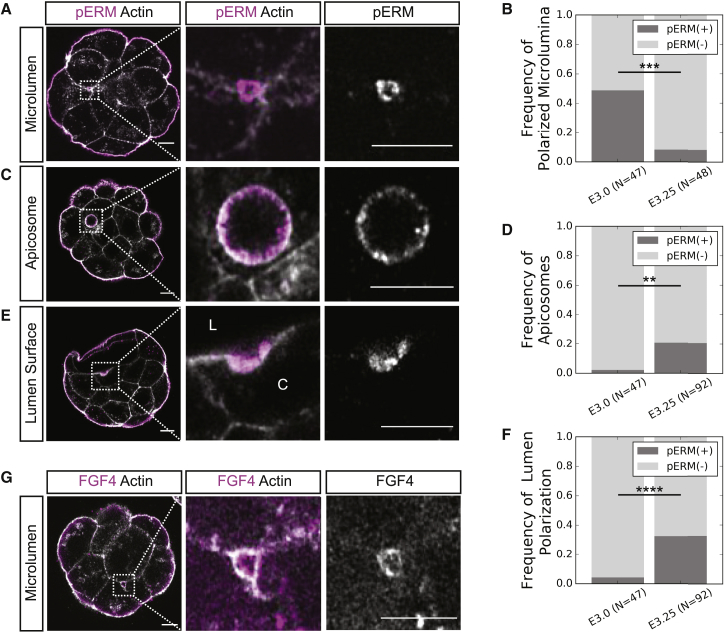
Microlumina Containing Secreted Apical Domain Components Are Transiently Upregulated during Early Phases of Fluid Accumulation (A) Representative immunofluorescence images of an apically polarized microlumina in an E3.0 embryo. (B) Frequency of apically polarized microlumina in E3.0 and E3.25 embryos (p < 0.001). (C) Representative immunofluorescence image of an E3.25 ICM cell containing an apicosome. (D) Frequency of apicosome occurrence in E3.0 and E3.25 embryos (p < 0.002). (E) Representative immunofluorescence image of an E3.25 ICM cell in which a subsection of its membrane facing the growing lumen is apically polarized (L-lumen; C-cytoplasm). (F) Frequency of lumen polarization in E3.0 and E3.25 embryos (p < 0.0001). (G) Z slice of an RNA-injected E3.0 embryo showing localization of FGF4-mNeonGreen to the membrane domains of a microlumen, representative of N = 7 embryos. All scale bars, 10 μm. Two-tailed Fisher's exact test ^∗∗∗∗^p < 0.0001, ^∗∗∗^p < 0.001, ^∗∗^p < 0.01 See also Figures S2 and S3; Videos S5 and S6; Table S2.

While the apico-basal polarity of TE cells surrounding the mouse blastocyst lumen is inverted to that of typical cysts and tubes (Alvers et al., 2014, Bryant et al., 2010, Bryant et al., 2014), the vesicle fusion observed in E3.0 embryos is similar to exocytosis of apical vacuolar compartments in apical cord hollowing, which is a *de novo* lumen formation mechanism that is conserved across species and tissues (Alvers et al., 2014, Bryant and Mostov, 2008, Sigurbjörnsdóttir et al., 2014). Critical to the initiation of apical cord hollowing is the formation of the apical membrane initiation site (AMIS) that dictates where the lumen will initiate and expand (Bryant et al., 2010, Ferrari et al., 2008). As such, we examined early lumen formation stage embryos for apical polarity phenotypes resembling reported AMIS and AMIS-like structures. Interestingly, we found that many E3.0 embryos contain microlumina enriched for the apical marker phosphorylated ERM (pERM) (43%, N = 20 of 47 embryos; Figures 2A and 2B). By E3.25 (90 h post-hCG), such structures are rare as the main lumen expands and individual microlumina merge with it (Figure 2B; p < 0.001, two-tailed Fisher's exact test). Although pERM localizes to microlumina, other apical lumen trafficking proteins, such as the small GTPase Rab11a (Alvers et al., 2014, Bagnat et al., 2007, Bryant et al., 2010, Bryant et al., 2014), are found in the subapical regions of TE cells instead of the cytoplasmic regions adjacent to microlumina (Figure S2A). Interestingly, we find that Integrin-β1 localizes to subpopulations of microlumina and nascently separated membrane domains (Figure S2B) exclusive of the pERM luminal structures (Figure S2C).

While apically polarized microlumina are infrequent in E3.25 embryos, we do observe the presence of two other apically polarized structures rarely found in E3.0 embryos (Figures 2C–2F). A small number of ICM cells in E3.25 embryos contain apicosome-like structures (21%, N = 19 of 92 embryos; Figures 2C and 2D; p < 0.002, two-tailed Fisher's exact test), which contain apical polarity proteins and have been proposed to be luminal precursors in human pluripotent stem cell culture (Taniguchi et al., 2017). Cells containing apicosome-like structures are isolated from contact-free surfaces created by the growing lumen (Figure 2C; Video S5). If such cells acquire sustained contact with the lumen the apicosome is released into the lumen over approximately 2–3 h post-contact (Video S6). Subsections of the lumen-facing membrane in a small number of E3.25 ICM cells express apical polarity markers and have a markedly shorter radius of curvature than that of the rest of the ICM-lumen interface, indicating recent fusion of either an apicosome or apically polarized microlumina (33%, N = 30 of 92 embryos; Figures 2E and 2F; p < 0.0001, two-tailed Fisher's exact test).

Video S5. Apicosome-like Structures Are Contained in Cells Isolated from Luminal Contact, Related to Figure 2 Z stack of a cell containing an apicosome-like structure (pERM, magenta; Actin, gray; nuclei, cyan). Scale bar, 5 μm.

Video S6. Apicosome-like Structures Are Released into Luminal Space When the Cell Gains a Contact-free Surface, Related to Figure 2 Time-lapse (time step = 15 min, hh:mm) of membrane signal (mT) in a cell releasing an apicosome-like structure into luminal space once the cell acquires a contact-free surface along the ICM-lumen interface. Scale bar, 10 μm.

FGF4 has been shown to be essential for PrE establishment (Kang et al., 2013, Krawchuk et al., 2013, Lanner and Rossant, 2010, Yamanaka et al., 2010), and its expression is restricted to EPI cells (E3.25–E4.5) (Frankenberg et al., 2011, Ohnishi et al., 2014). Because FGF ligands have been shown to create signaling niches by localizing to microlumina (Durdu et al., 2014), we examined the localization of FGF4 protein in E3.0 embryos by injecting mRNA of *fgf4-mNeonGreen* into a single blastomere of a 4-cell stage embryo. We observe localization of FGF4-mNeonGreen on the membranes of a subset of microlumina (36%, N = 7 embryos, Figure 2G), while signal in the cytoplasm is significantly lower (Figure S3A) and *mNeonGreen* without the *fgf4* coding sequence does not localize to microlumina (Figure S3B). The localization of FGF4 to microlumina suggests that luminal microenvironments may provide a signaling cue capable of influencing fate specification in surrounding cells.

### ICM Spatial Patterning Resolves as the Lumen Expands

During the final phases of coalescence and lumen expansion, initial transcriptional biases of ICM cells toward either EPI or PrE fate are either reinforced or changed concomitantly with spatial segregation of the lineages (Chazaud et al., 2006, Frankenberg et al., 2011, Ohnishi et al., 2014, Plusa et al., 2008). Cells that are molecularly specified to become PrE but fail to achieve correct positioning along the ICM-lumen interface in a timely manner have been proposed to undergo apoptosis (Plusa et al., 2008). However, it is not known precisely when the cells begin to undergo repositioning since initial position of the precursors is stochastic (Chazaud et al., 2006, Gerbe et al., 2008), and lumen formation onset is variable between embryos in terms of developmental time. To determine whether spatial segregation is correlated with luminal volume, we used lineage reporters and membrane signal (Muzumdar et al., 2007) to track the emergence and positioning of EPI (Arnold et al., 2011) and PrE precursor cells (Hamilton et al., 2003) within the ICM in relation to the expanding lumen.

**Figure 3:**
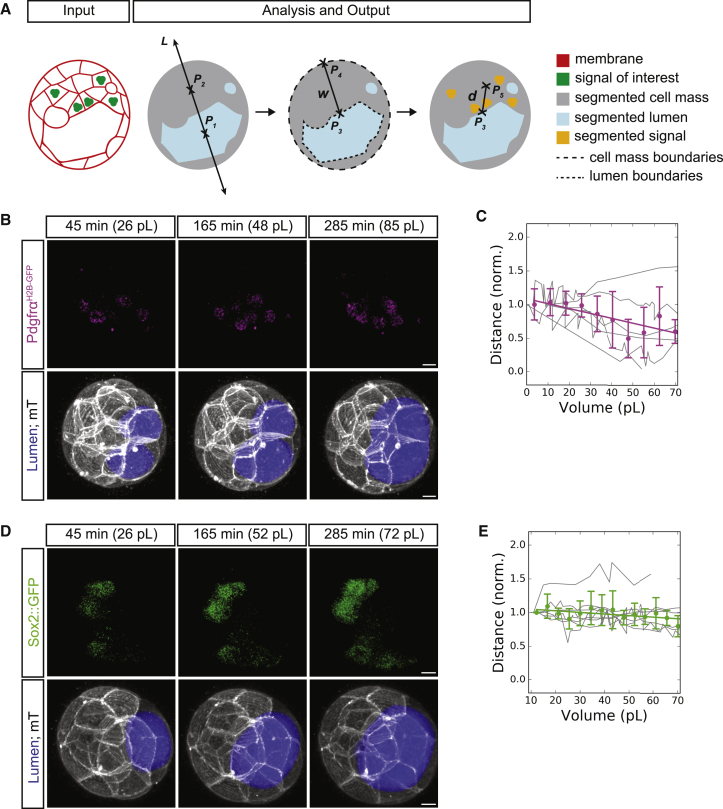
The Pdgfr⍺ Signaling Domain Approaches the Luminal Surface as the Lumen Grows in Volume (A) Schematic 2D representation of 3D analysis method for the tracking and normalization of fate reporter expression proximity to the ICM-lumen interface. $P_{1,2,3,4,5}$ are 3D points. $\overset{↔}{L}$ is a 3D line ($\overset{↔}{P_{1}P_{2}}$ equivalent) that defines the embryonic-abembryonic axis. $\bar{w}$ is the 3D line segment ($\bar{P_{3}P_{4}}$ equivalent) that measures the ICM width. $\bar{d}$ is the 3D line segment ($\bar{P_{3}P_{5}}$ equivalent) that measures the distance from the center of mass of the signal of interest to the ICM-lumen interface. See Image Analysis for formal definitions of all geometric entities. (B) Time-lapse of an E3.0 embryo expressing a PrE reporter (*Pdgfr⍺*^*H2B-GFP/+*^; top), a membrane marker and lumen segmentation (bottom). *t* = 0 min is defined as the first moment when a lumen can be segmented. Lumen volume (pL) is given for each time point shown. (C) Quantification of the distance of the center of Pdgfr⍺ signaling domain to the surface of the lumen over time (N = 6 embryos, thin gray lines are traces of individual embryos, magenta dots are binned averages with vertical capped lines showing standard deviations, thick magenta line is the linear regression y = −0.007x + 1.090, r^2^ = 0.653, p < 0.005). (D) Time-lapse of an E3.0 embryo expressing a cytoplasmic EPI reporter (*Sox2::gfp*; top), a membrane marker and lumen segmentation (bottom). *t*_*0*_ is defined as the first moment when a lumen can be segmented. Lumen volume (pL) is given for each time point shown. (E) Quantification of the distance of the center of Sox2 expression domain to the surface of the lumen over time (N = 8 embryos, thin gray lines are traces of individual embryos, green dots are binned averages with vertical capped lines showing standard deviations, thick green line is the linear regression y = −0.002x + 1.074, r^2^ = 0.339, p < 0.030). All scale bars, 10 μm.

In order to measure and track the EPI and PrE domains, we developed an analysis method that combines gross morphological changes within the embryo with tissue molecular identity and position during lumen formation and expansion. First, we simultaneously segment the lumen and embryo cell mass to determine the embryonic-abembryonic axis, which provides robust spatial orientation (Figure 3A; see STAR Methods for details). The resulting method allows us to determine the distance of each ICM lineage from the ICM-lumen interface relative to the volume of the expanding lumen and the changing morphology of the ICM.

The EPI reporter (Sox2::gfp) center showed no net movement toward the ICM-lumen interface and maintained a relatively constant distance from the luminal surface throughout expansion (Figures 3D and 3E). In contrast, the PrE reporter (Pdgfr⍺^H2B-GFP/+^) center shifts toward the ICM-lumen interface as soon as coalescence begins (approximately E3.25) and continues throughout expansion (Figures 3B and 3C). This indicates differential sorting behavior between PrE and EPI cells and suggests that lumen expansion may play a role in guiding EPI-PrE fate specification and spatial segregation.

### ICM Lineage Specification and Spatial Segregation Are Dependent on Luminal Expansion

**Figure 4:**
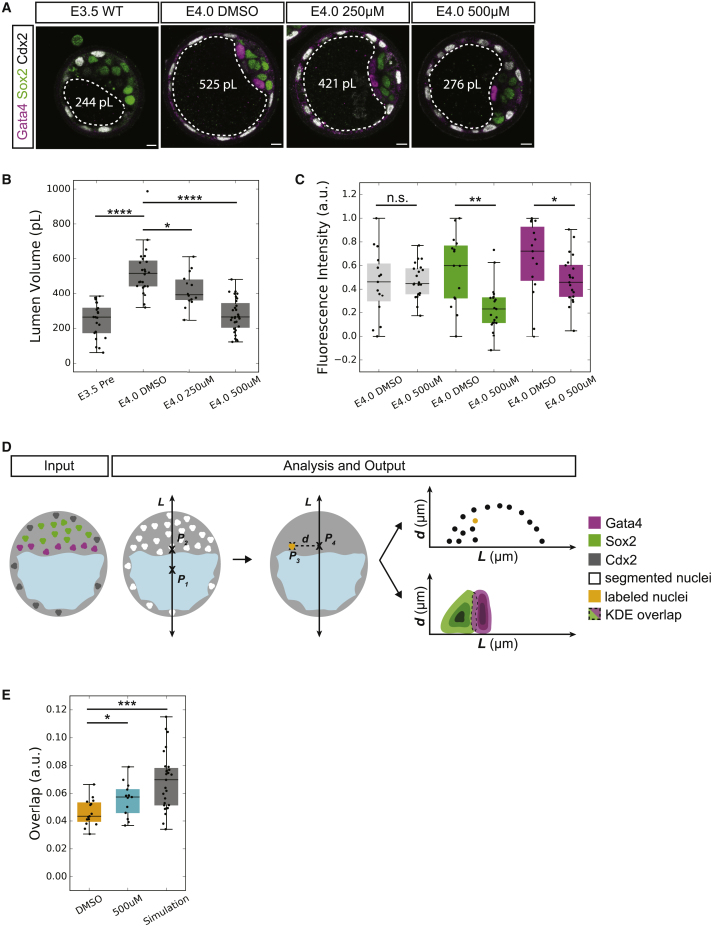
EPI and PrE Expression Levels Are Reduced in ATP1-Inhibited Embryos (A) Immunofluorescence images of TE (Cdx2), EPI (Sox2), and PrE (Gata4) fate in pre-treatment control (E3.5 WT), Atp1 inhibited (E4.0 500 μM and E4.0 250 μM), and end-stage control (E4.0 DMSO) embryos. Lumen boundaries outlined by dashed white line and mean lumen volume in white text. Scale bars, 10 μm. (B) Boxplot of lumen volume for E3.5 WT (N = 21), E4.0 DMSO (N = 24), E4.0 250 μM Atp1 inhibited (N = 14) and E4.0 500 μM Atp1 inhibited (N = 31) embryos indicating that the impact on lumen volume is concentration dependent. (C) Boxplot of fluorescence levels of Cdx2 (gray), Sox2 (green), and Gata4 (magenta) in E4.0 500 μM Atp1 inhibited embryos compared to E4.0 DMSO controls. (D) Schematic 2D representation of 3D analysis method for spatial segregation of ICM lineages. $P_{1,2,3,4}$ are 3D points. $\overset{↔}{L}$ is a 3D line ($\overset{↔}{P_{1}P_{2}}$ equivalent) that defines the embryonic-abembryonic axis. $\bar{d}$ is the 3D line segment ($\bar{P_{3}P_{4}}$ equivalent) that measures the perpendicular distance from the center of a cell to $\overset{↔}{L}$. See Image Analysis for formal definitions of all geometric entities. (E) Boxplot of spatial overlap between EPI and PrE lineages within E4.0 control (DMSO, N = 15), E4.0 Atp1 inhibited (500 μM, N =13) and simulated data of maximal overlap in E4.0 WT embryos (Simulation, N = 27). ^∗∗∗∗^p < 0.0001, ^∗∗∗^p < 0.001, ^∗∗^p < 0.01, ^∗^p < 0.05. n.s., not significant. For boxplots: central mark indicates the median; lower edge, 25%; upper edge, 75%; lower whisker, Q1 − (1.5 × IQR), where IQR = Q3 − Q1; upper whisker, Q3 + (1.5 × IQR). See also Figures S4 and S5; Table S2.

Given the correlation between lumen expansion and the oriented movement of PrE progenitor cells (Figures 3D and 3E), we hypothesized that modulation of lumen size may impact lineage differentiation and cell positioning. To test this hypothesis, we inhibited the expansion of post-coalescence stage lumina (96–108 h post-hCG; E3.5–E4.0) through multiple means. Embryos cultured in media containing 500 μM ouabain (Wiley, 1984) show a significant decrease in luminal volume. The volume effect can be modulated by changing inhibitor concentration (Figures 4A, 4B, and S4A), suggesting that the effect of ouabain in reducing lumen expansion is specifically due to its action on the Atp1 channel. The inhibition of the cystic fibrosis transmembrane conductance regulator, a Cl^−^ channel known to impact lumen expansion in other systems (Bagnat et al., 2010, Navis and Bagnat, 2015a, Navis and Bagnat, 2015b), also results in reduced lumen size, although the reduction is not as significant as Atp1 inhibition (Figure S4C). Notably, Atp1 inhibited embryos show significant reduction in the fluorescence levels of both EPI and PrE markers (Figure 4C) in addition to possessing a significantly lower luminal volume than that of controls without a change in the total number of cells (Figure S4B).

To determine if the spatial segregation of EPI and PrE is perturbed in Atp1 inhibited embryos, we developed a 3D analysis method that integrates position and molecular information. Using this, EPI-PrE overlap is defined as the intersection of the probability of the two populations based on spatial position in 3D and the fluorescence levels of Sox2 (EPI) and Gata4 (PrE) (Figure 4C; see STAR Methods for details). No spatial segregation would result in maximum overlap, which is defined and simulated as the scenario in which all ICM cells are equally likely to be EPI or PrE (“Simulation,” mean_Simulation_ = 0.069; maximum_Simulation_ = 0.115; minimumE4.0_WT_ = 0.0002). This analysis shows that in accordance with the hypothesis, Atp1 inhibited embryos indeed have a significantly higher degree of overlap between EPI and PrE populations (Figure 4D; mean_DMSO_ = 0.046, mean_500__μM_ = 0.057, p < 0.036), indicating that the spatial segregation of ICM lineages is dependent on luminal expansion.

**Figure 5:**
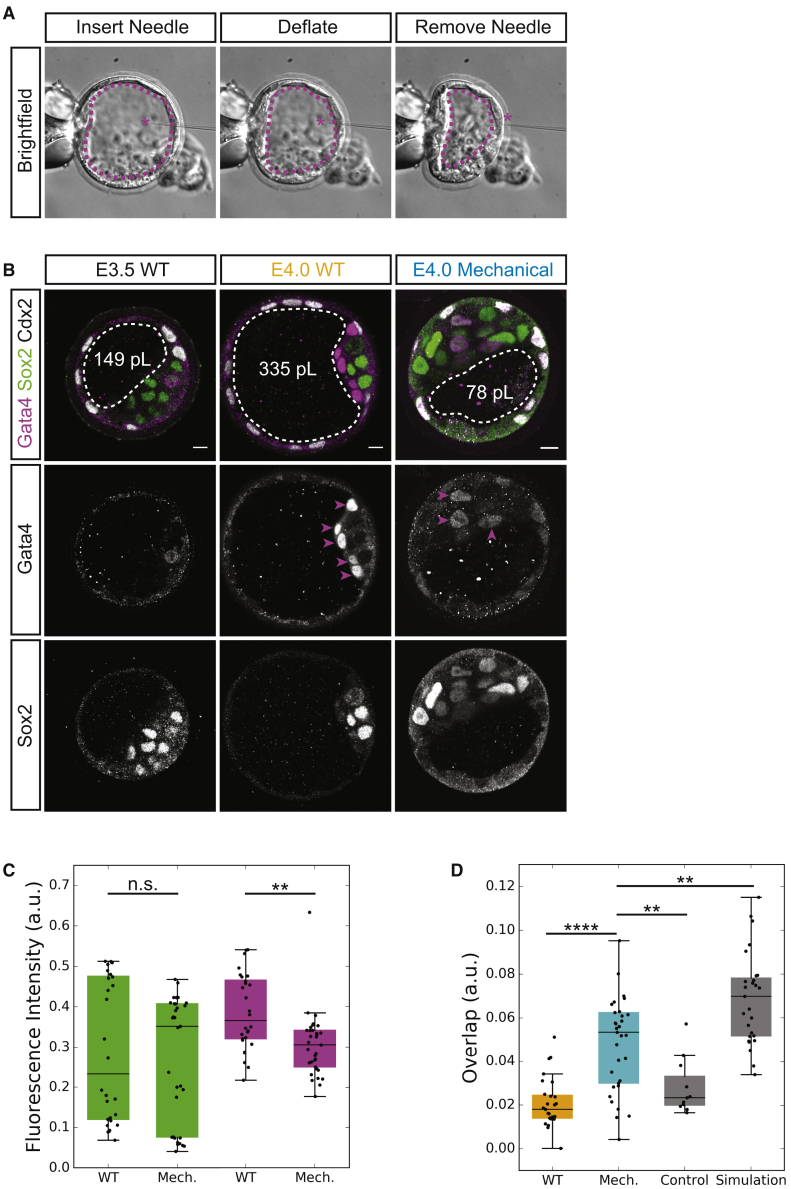
PrE Specification and Spatial Segregation of ICM Lineages Is Impaired by Mechanical Inhibition of Lumen Expansion (A) Brightfield images of mechanical deflation. Magenta asterisk marks the needle tip. Dotted magenta line indicates lumen boundary. (B) Immunofluorescence images of EPI (Sox2) and PrE (Gata4) fate in pre-manipulation control (E3.5 WT), E4.0 post-manipulation control (E4.0 WT), and E4.0 mechanically inhibited (E4.0 Mechanical) embryos. Magenta arrowheads indicate the position of cells expressing high levels of Gata4 within the ICM. White dotted line indicates lumen boundaries. Average lumen volume in white text. Scale bars, 10 μm. (C) Boxplot of fluorescence levels of Sox2 (green) and Gata4 (magenta) in mechanically inhibited (Mech., N = 33) and post-manipulation control (WT, N = 28) E4.0 embryos. (D) Boxplot of spatial overlap between EPI and PrE lineages within post-manipulation control (WT, N = 27), mechanically inhibited (Mech., N = 33), E4.0 procedural control (Control, N = 11), and E4.0 simulation of complete overlap in WT conditions (Simulation, N = 27). ^∗∗∗∗^p < 0.0001, ^∗∗^p < 0.01. n.s., not significant. For boxplots: central mark indicates the median; lower edge, 25%; upper edge, 75%; lower whisker, Q1 − (1.5 × IQR), where IQR = Q3 − Q1; upper whisker, Q3 + (1.5 × IQR). See also Figure S6 and Table S2.

To further examine the possible role of lumen expansion in cell fate specification and sorting, we mechanically deflated late expansion stage lumina (E3.5–E4.0) by inserting a microneedle into the lumen at the junctions of mural TE cells and applying negative pressure to counteract expansion (Figure 5A). This action was repeated every 1–2 h as necessary for individual embryos so that the blastocyst lumen volume did not change significantly from that of the initial E3.5 blastocysts. To ensure observations are not due to adverse effects induced by serial needle insertion, we performed a control of passive lumen deflation due to serial puncture (“Control”) with no application of negative pressure. Importantly, serial needle insertion does not perturb tissue fidelity of lineage markers or embryo cell cycle progression (Figures S6A–S6C).

Interestingly, PrE specification levels are significantly reduced by mechanical deflation, while EPI specification levels are maintained at the level of WT embryos (Figures 5B and 5C). Furthermore, mechanical deflation impairs the spatial segregation of EPI and PrE cells (Figures 5B and 5D). EPI-PrE overlap of mechanically deflated embryos is more than doubled in comparison to WT controls (mean_Mech_ = 0.053, mean_WT_ = 0.021, p < 0.0001), but is still significantly less than that of simulated maximal overlap values (“Simulation,” p < 0.005). Spatial segregation analysis of controls shows a slightly higher degree of overlap between EPI and PrE domains in comparison to that of WT (mean_Control_ = 0.028, p < 0.045) while still being significantly lower than that of mechanically deflated embryos (p < 0.007).

Combined, the results of pharmacological and mechanical inhibition consistently show that timely lumen expansion facilitates fate specification and spatial segregation of EPI-PrE cell lineages.

### Potential Role of Luminal FGF4 in EPI-PrE Specification

**Figure 6:**
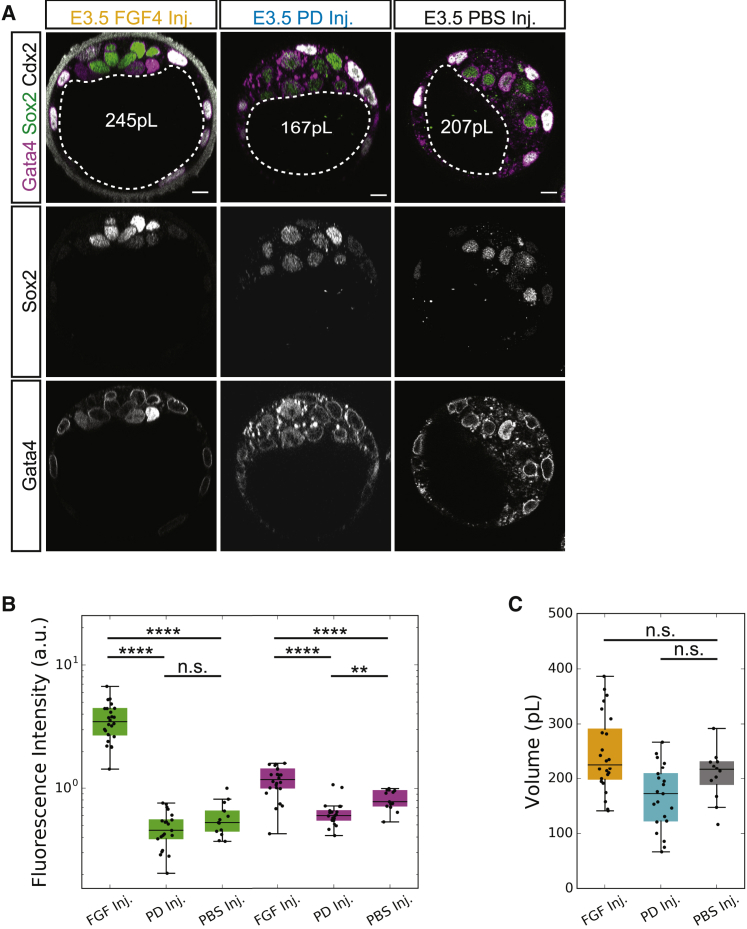
Perturbation of FGF4 Signaling in the Lumen Impacts Molecular Specification of EPI and PrE Lineages (A) Immunofluorescence images of EPI (Sox2) and PrE (Gata4) fate in E3.5 post-FGF4 deposition (E3.5 FGF4 Inj.), E3.5 post-PD173074 deposition (E3.5 PD Inj.), and E3.5 post-PBS deposition (E3.5 PBS Inj.). White dotted line indicates lumen boundaries. Average lumen volume in white text. Scale bars, 10 μm. (B) Boxplot of fluorescence levels of Sox2 (green) and Gata4 (magenta) in E3.5 post-FGF4 deposition (FGF4 Inj., N = 24), E3.5 post-PD173074 deposition (PD Inj., N = 21), and E3.5 post-PBS deposition (PBS Inj., N = 13) embryos. (C) Boxplot of luminal volume in E3.5 post-FGF4 deposition (FGF4 Inj., N = 24), E3.5 post-PD173074 deposition (PD Inj., N = 21), and E3.5 post-PBS deposition (PBS Inj., N = 13). ^∗∗∗∗^p < 0.0001, ^∗∗^p < 0.01. n.s., not significant. For boxplots: central mark indicates the median; lower edge, 25%; upper edge, 75%; lower whisker, Q1 − (1.5 × IQR) where IQR = Q3 − Q1; upper whisker, Q3 + (1.5 × IQR). See also Figure S7.

As pharmacological and mechanical deflation experiments cannot distinguish the mechanical and biochemical influences of the lumen on EPI-PrE development, we next altered the luminal contents in embryos undergoing early expansion (E3.0–E3.5), without affecting the luminal volume. To investigate the potential role of the lumen as a signaling niche, we chose to enhance or inhibit FGF4 signaling through luminal deposition (see STAR Methods) of FGF4 protein or an FGFR1 inhibitor (Yamanaka et al., 2010), respectively; PBS deposition was used as a control. Embryos with luminally deposited FGF4 (FGF4-I; 500 ng/mL FGF4 with 1 μg/mL heparin) exhibit Gata4 and Sox2 expression levels significantly higher than that of control embryos (Figures 6A and 6B). In comparison, embryos with luminally deposited PD173074 (PD-I; 200 nM) exhibit significantly lower Gata4 levels than that of control (Figure 6B). Note that the differences in luminal volume between FGF4-I and PD-I embryos with that of the PBS-I control embryos is not significant (Figure 6C), suggesting that the impact on cell fate is driven by changes in luminal contents rather than changes in lumen size. The number of cells in the embryo also remains unchanged (Figure S7). While the reduction of Gata4 levels of PD-I embryos is similar to the changes reported in globally inhibited embryos (Yamanaka et al., 2010), the increased expression levels of both Sox2 and Gata4 in FGF-I embryos differs from global treatments, which show conversion of the entire ICM to PrE at the expense of the EPI population (Kang et al., 2013, Yamanaka et al., 2010). The increased expression of both EPI and PrE markers in FGF4-I embryos suggests the importance of local availability of fate specifying factors within the embryo.

**Figure 7:**
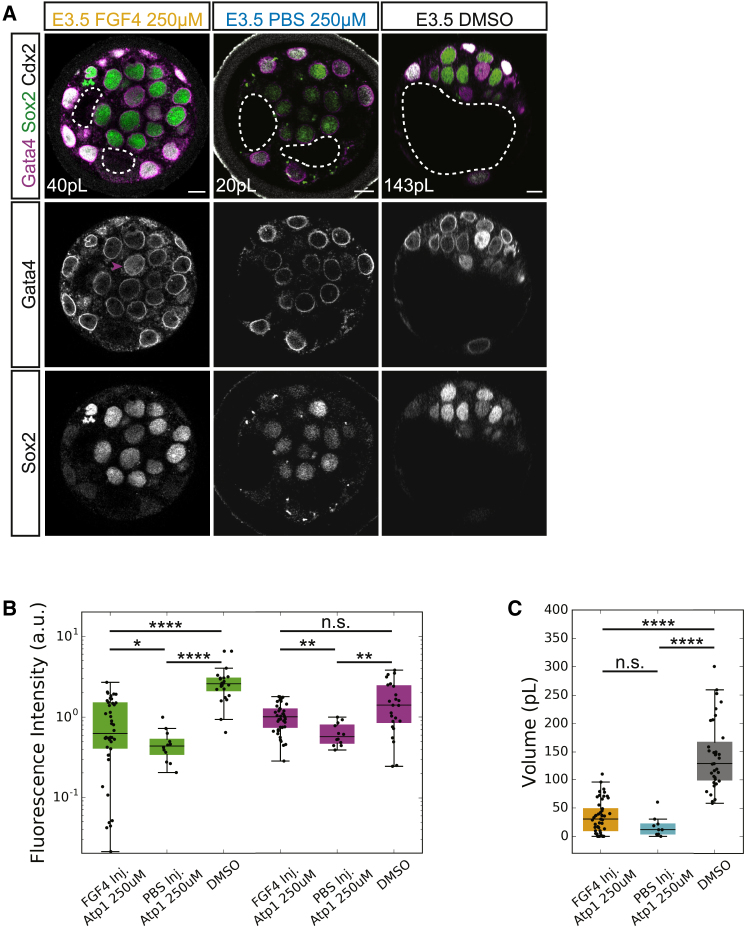
Luminal Deposition of FGF4 Partially Rescues EPI-PrE Specification in ATP1-Inhibited Embryos (A) Immunofluorescence images of EPI (Sox2) and PrE (Gata4) fate in E3.5 post-FGF4 deposition and Atp1 inhibition (E3.5 FGF4 250 μM), E3.5 post-PBS deposition and Atp1 inhibition (E3.5 PBS 250 μM), and E3.5 control embryos (E3.5 DMSO). White dotted line indicates lumen boundaries. Average lumen volume in white text. Magenta arrowhead indicates cell with high Gata4 expression relative to neighboring cells. Scale bars, 10 μm. (B) Boxplot of fluorescence levels of Sox2 (green) and Gata4 (magenta) in E3.5 post-FGF4 deposition and Atp1 inhibition (250 μM FGF4 Inj., N = 42 embryos), E3.5 post-PBS deposition and Atp1 inhibition (250 μM PBS Inj., N = 12), and E3.5 control embryos (DMSO, N = 12). (C) Boxplot of luminal volume in E3.5 post-FGF4 deposition and Atp1 inhibition (250 μM FGF4 Inj., N = 49 embryos), E3.5 post-PBS deposition and Atp1 inhibition (250μM PBS Inj., N = 9), and E3.5 control embryos (DMSO, N = 38). ^∗^p < 0.05, ^∗∗^p < 0.01, ^∗∗∗∗^p < 0.0001. n.s., not significant. For boxplots: central mark indicates the median; lower edge, 25%; upper edge, 75%; lower whisker, Q1 − (1.5 × IQR), where IQR = Q3 − Q1; upper whisker, Q3 + (1.5 × IQR).

To further dissect the mechanical or biochemical role of the lumen, we tested if luminal deposition of FGF4 can rescue the reduced EPI-PrE molecular specification levels in embryos with reduced lumen size (Figures 4 and 5). We performed FGF4 luminal deposition and cultured the injected embryos in media containing ouabain (FGF4-OUA; 250 μM), the Atp1 inhibitor previously used (Figures 1 and 4). PBS deposition and subsequent Atp1 inhibition (PBS-OUA) was used as a control in addition to standard DMSO controls. FGF4-OUA embryos show a significant increase in the specification of both EPI (Sox2) and PrE (Gata4) lineages in comparison with that of PBS-OUA controls (Figures 7A and 7B). These changes in cell fate specification are decoupled from lumen expansion given that FGF4-OUA luminal volume is comparable to that of PBS-OUA (Figure 7C). These results show that luminal deposition of FGF4 can partially rescue EPI-PrE specification in embryos with reduced lumen expansion. Taken together, our findings reveal a potential biochemical function of the lumen to impact ICM lineage specification and maturation.

## Discussion

Collectively, the data presented here reveal a mechanism of blastocyst lumen formation and its role in facilitating the specification and positioning of the ICM lineages. We have shown that all cells of the embryo, not only the trophectoderm, can contribute to lumen initiation and growth through a vesicle secretion mechanism (Figure 1), in agreement with previous studies (Wiley and Eglitis, 1980, Wiley and Eglitis, 1981, Fleming and Pickering, 1985, Aziz and Alexandre, 1991). The apicosome-like structures observed in E3.25 embryos (Figure 2C) show that ICM cells isolated from contact-free surfaces can generate luminal precursor-like structures (Taniguchi et al., 2015, Taniguchi et al., 2017), which is in line with our observation that all cells of the embryo contribute to lumen formation.

The presence of apically polarized microlumina in E3.0 embryos (Figure 2A) along with the widespread vesicle release (Figure 1C) reveal notable parallels between blastocyst lumen formation mechanisms and apical cord hollowing. Furthermore, the morphology and secretion dynamics of the vesicles reported here are markedly similar to those present in systems that require regulated secretion of contents from large vesicles into a lumen (Miklavc et al., 2012, Rousso et al., 2016, Segal et al., 2018, Tran et al., 2015). However, the localizations of Rab11 (Figure S2A) and Integrin-β1 (Figures S2B and S2C) during the onset of fluid accumulation suggest that the molecular mechanisms of blastocyst lumen formation are differentially regulated than that of typical apical cord hollowing. If molecules such as morphogens or chemokines are secreted through this mechanism, this could provide a causal link between fluid accumulation and fate specification and/or cell migration (Durdu et al., 2014). In addition, if vesicles contain highly concentrated osmolytes or cell-adhesion modifying molecules (e.g., Integrin-β1 or extracellular matrix cofactors; Figures S2B and S2C), secretion would facilitate downstream lumen expansion as seen in other systems (Takeda et al., 2000, Strilić et al., 2010).

We have shown that immediately following coalescence of microlumina into a singular lumen, PrE-biased ICM cells begin directional movement toward the ICM-lumen interface (Figure 3). When lumen expansion is perturbed, PrE maturation and repositioning is disrupted (Figures 4 and 5), suggesting a causal role of lumen expansion in guiding EPI-PrE cell fate specification and spatial sorting, which is crucial for tissue organization in downstream embryonic development (Moore et al., 2014, Morris et al., 2002, Yang et al., 2007). These results indicate that spatial segregation of ICM cell lineages initiates earlier than previously thought (Chazaud et al., 2006, Gerbe et al., 2008) and depends on morphological changes within the embryo.

The net movement of PrE cells to the luminal surface in accordance with lumen expansion (Figures 3, 4, and 5) is reminiscent of chemokine-guided migration (Boldajipour et al., 2008, Bussmann and Raz, 2015, Donà et al., 2013). Spatial segregation between PrE and EPI cells suggests their differential reception of luminal cues (Figure 3). The presence of FGF4 in microlumina (Figure 2), its expediting effect on fate specification when luminally deposited (Figure 6), and partial rescue of the impact of reduced luminal volume (Figure 7) suggest an instructive role of FGF4 accumulated in the lumen. One such scenario is that as the lumen expands, luminal FGF4 concentration increases to guide ICM differentiation. While future studies to profile the luminal contents will be necessary to explore this hypothesis in depth, we have taken a first attempt to decouple the potential biochemical and mechanical roles of the lumen in EPI-PrE specification (Figures 6 and 7).

In addition to signaling molecules present in the fluid, the constantly changing physical microenvironment within the blastocyst on account of lumen emergence, coalescence, and expansion may also impact tissue remodeling through changes in cell adhesion and cell shape (Dumortier et al., 2019, Durdu et al., 2014, Engler et al., 2006, Mammoto et al., 2013). It has been shown that in epithelial systems multi-luminal phenotypes due to incomplete coalescence can alter the ratios of cell types within tissues and disrupt tissue function (Bagnat et al., 2007, Bryant et al., 2010, Chou et al., 2016, Kesavan et al., 2009). Recent work shows that lumen expansion plays an important role in controlling embryo size and spatial allocation of TE and ICM cells (Chan et al., 2019). In this study, we extend this finding to the second lineage segregation event and found that, in addition to its mechanical role, the lumen can also provide a signaling cue that could affect EPI-PrE specification within the ICM.

Taken together, these data suggest that lumen expansion may provide a niche in which chemical and physical cues are integrated to guide ICM self-organization in the blastocyst. Further studies will need to be conducted in order to understand the mechanisms and interplay underlying lumen formation, cell polarization, and fate specification during blastocyst morphogenesis.

## STAR★Methods

### Key Resources Table

**Table:** 

Reagent or Resource	Source	Identifier
**Antibodies**		

Goat anti-Sox2	Santa Cruz Biotechnology	sc-17320; RRID: AB_2286684
Rabbit anti-Sox2	Cell Signaling Technologies	23064; RRID: AB_2714146
Goat anti-Sox2	R&D Systems	AF2018; RRID: AB_355110
Mouse anti-Oct3/4	Santa Cruz Biotechnology	sc-5279; RRID: AB_628051
Rabbit anti-Gata4	Santa Cruz Biotechnology	sc-9053; RRID: AB_2247396
Goat anti-Gata4	R&D Systems	AF2606-SP; RRID: AB_2232177
Goat anti-Biotinylated Gata4	R&D Systems	BAF2606; RRID: AB_2263176
Mouse anti-Cdx2	BioGenex	MU392A-UC; RRID: AB_2650531
Rabbit anti-pERM	Cell Signaling Technologies	3726; RRID: AB_10560513
Mouse anti-Rab11	BD Biosciences	610656; RRID: AB_397983
Rat anti-Integrin-β1	Merck Millipore	MAB1997; RRID: AB_2128202
Rabbit anti-GFP	MBL	598; RRID: AB_591816
Mouse anti-Hsp47	Enzo Life Sciences	M16.10A1; RRID: AB_10618557

**Chemicals, Peptides, and Recombinant Proteins**		

Pregnant mare’s serum gonadotropin	Intervet	Intergonan
Human chorionic gonadotropin	Intervet	Ovogest 1500
KSOMaa	Zenith biotech	ZEKS-050
KSOMaa with Hepes	Zenith biotech	ZEHP-060
BSA	Sigma	A3311
Mineral oil	Sigma	M8410
Ouabain	Sigma	O3125
Brefeldin A	Sigma	B6542
CFTR Inhibitor-172	Santa Cruz Biotechnologies	sc-204680
DMSO (Dimethyl sulfoxide)	Sigma	D2650
Recombinant Human FGF4 Protein	R&D Systems	235-F4-025
PD173074	Torcis	3044
Heparin	Sigma	H3393

**Critical Commercial Assay**		

mMessage mMachine™ transcription kit	Invitrogen/ ThermoFisher Scientific	AM1340M
MEGAscript™ T7 Transcription Kit	Invitrogen/ Thermo Fisher Scientific	AM1344

**Experimental Models: Organisms/Strains**		

Mouse: (C57BL/6xC3H) F1	Laboratory Animal Resources at the European Molecular Biology Laboratory	N/A
Mouse: mTmG	The Jackson Laboratory; Muzumdar et al., 2007	007676
Mouse: Lifeact-GFP	Riedl et al., 2010	N/A
Mouse: Pdgfr⍺^H2B-GFP^	Hamilton et al., 2003	N/A
Mouse: Sox2::GFP	Arnold et al., 2011	N/A

**Software and Algorithms**		

FIJI	Schindelin et al., 2012	https://fiji.sc
AutoFocusScreen	Rabut and Ellenberg, 2004	N/A
Python	N/A	https://www.python.org/
Custom Lumen and Tissue Segmentation Functions	This Study	https://github.com/allysonryan/phd_notebooks.git

**Other**		

μ-Slide Angiogenesis chambered coverslip	MatTek	P35G-1.5-14-C

### Lead Contact and Materials Availability

Further information and requests for resources and reagents should be directed to and will be fulfilled by the Lead Contact, Takashi Hiiragi (hiiragi@embl.de). All unique/stable reagents generated in this study are available from the Lead Contact with a completed Materials Transfer Agreement.

### Experimental Model and Subject Details

#### Animal Work

All animal work was performed in the Laboratory Animal Resources (LAR) Facility at European Molecular Biology Laboratory (EMBL) with permission from the institutional veterinarian (ARC number TH110011). LAR Facilities operate according to international animal welfare guidelines (Federation for Laboratory Animal Science Associations guidelines and recommendations). All experimental mice were maintained in specific pathogen-free conditions on a 12–12-hr light-dark cycle and used from 8 weeks of age.

#### Transgenic Mice and Genotyping

The following mouse lines were used in this study: (C57BL/6xC3H) F1 as WT, mTmG (Muzumdar et al., 2007), Lifeact-EGFP (Riedl et al., 2010), Pdgfr⍺^H2B-GFP^ (Hamilton et al., 2003) and Sox2-GFP (Arnold et al., 2011). Standard tail genotyping procedures were used to genotype mice (for primers and PCR product sizes see Table S1).

#### Mouse Embryo Recovery and Culture

To obtain pre-implantation embryos, female mice were superovulated by intraperitoneal injection of 5 or 7.5 international units (IU) of pregnant mare’s serum gonadotropin (Intervet, Intergonan) followed by 5 or 7.5 IU of human chorionic gonadotropin (hCG; Intervet, Ovogest 1500) 48 hours later. Hormone injection dosage was batch dependent and determined by LAR services. Superovulated females were mated with male mice directly after hCG injection. Embryos were flushed from dissected oviducts and uteri of female mice after super-ovulation and mating with male mice. Embryos were flushed from dissected oviducts and uteri at 48, 72, 78, 84, 96 and 108 hours post-hCG using KSOMaa with Hepes (Zenith Biotech, ZEHP-060). After flushing, embryos were washed in KSOMaa with Hepes, transferred to 10μL drops of KSOMaa (Zenith Biotech, ZEHP-050) covered with mineral oil (Sigma, M8410) on either a tissue culture dish (Falcon, 353001) or petri dish (Falcon, 351008) and then cultured at 37^°^C in a CO_2_ incubator (Thermo Scientific, Heracell 240i) with 5% CO_2_.

### Method Details

#### Pharmacological Inhibition

Ouabain (Sigma, O3125) was resuspended in DMSO (Sigma, D2650) at a stock concentration of 100mM. For working concentrations of 500μM, 250μM and 100μM the stock concentration was diluted in KSOMaa. Brefeldin A (Sigma, B6542) was resuspended in DMSO at a stock concentration of 1.4M. For working concentrations of 14μM and 7μM the stock concentration was diluted in KSOMaa. CFTR Inhibitor-172 (Santa Cruz Biotechnologies, sc-204680) was resuspended in DMSO at a stock concentration of 1mM. For working concentrations of 10μM and 5μM the stock concentration was diluted in KSOMaa. Embryos were incubated with the appropriate working concentration of ouabain, brefeldin A, CFTR Inhibitor-172 or an equivalent DMSO concentration in μ-Slide chambered coverslips (Ibidi, 81506) for a 12-hr period before fixation in 4% PFA (see Immunofluorescence Staining).

#### Serial Mechanical Deflation

Embryos were mounted on epifluorescence microscope (Zeiss, Observer.Z1) equipped with temperature-controlled incubation chamber and visualized using transmitted light. A micromanipulator (Narishige, MON202-D) with a glass holding needle (Harvard Apparatus, GC100T-15) was used to stabilize the embryo while a fine-tipped glass needle attached to a second micromanipulator was used to actively deflate the lumen by penetrating the mural TE at the junction of two cells and manually applying negative pressure. This action was repeated multiple times during the experimental window.

As a procedural control, the deflation needle was inserted at the junction of two mural TE cells in the same manner as experimental embryos and subsequently removed while maintaining a net zero difference in pressure between the lumen and the deflation needle throughout the entire duration of the procedure.

#### Luminal Deposition

Embryos were mounted and stabilized in the same fashion as for Serial Mechanical Deflation (see above). An injection needle containing either a recombinant FGF4 protein solution (500ng/mL FGF4 with 1μg/mL heparin; R&D Systems, 235-F4-025; Sigma, H3393), an FGFR1 inhibitor solution (200nM PD173074; Torcis, 3044) or PBS (as a control) was inserted between mural TE cells into a developing lumen. Positive pressure was then applied to deposit the solution in the lumen.

#### Cloning and *In Vitro* Transcription

The CDS of *FGF4* without a stop codon was cloned into a Gateway middle entry clone and then used in an LR reaction with a 5′ entry clone containing an SP6 site and a 3′ entry clone containing the CDS of *mNeonGreen* with a polyA site. Standard pGEM cloning was used to create a control plasmid with mNeonGreen with a T7 site.

#### mRNA Injection

Linearized plasmid or cleaned PCR product was used as the template for an *in vitro* transcription reaction from an Invitrogen mMessage SP6 kit (AM1340M) or an Invitrogen MEGASCRIPT T7 kit (AM1334). mRNA injections were performed on the same microscope and with the same micromanipulators as described earlier (see Serial Mechanical Deflation). mRNA was injected into a single blastomere of 4-cell stage embryos at a concentration of 200ng/μl using a needle (Harvard Apparatus, G100TF-15) attached to an injector (Eppendorf, FemtoJet).

#### Immunofluorescence Staining

Embryos were fixed with 4% PFA for 15 minutes at room temperature and subsequently permeabilized with PBS (0.5% Triton-X) for 20 minutes at room temperature before transferring to blocking buffer (PBS with 0.1% Tween-20; 5% BSA) for at least 4hrs at 4^°^C. Embryos were incubated with primary antibodies diluted in blocking buffer overnight at 4^°^C. After washing with blocking buffer, embryos were incubated with secondary antibodies diluted in blocking buffer for 2hrs at room temperature. Finally, embryos were rinsed with PBS before being mounted in a DAPI solution (PBS with 1:2000 DAPI; Invitrogen, D3571) for imaging (see Fixed Sample Imaging).

The following primary antibodies were used in this study: rabbit anti-pERM (Cell Signaling, 3726), mouse anti-Cdx2 (BioGenex, MU392A-UC), goat anti-Sox2 (Santa Cruz Biotechnology, sc-17320), goat anti-Sox2 (R&D Systems, AF2018-SP), rabbit anti-Sox2 (Cell Signaling, 23064), rabbit anti Gata4 (Santa Cruz Biotechnology, sc-9053), goat anti-Gata4 (R&D Systems, AF2606-SP), rabbit anti-GFP (MBL, 598), mouse anti-Rab11 (BD Biosciences; 610656), rat anti-integrin-β1 (Merck Millipore, MAB1997), goat anti-biotinylated Gata4 (R&D Systems, BAF2606) mouse anti-Oct3/4 (Santa Cruz Biotechnology, sc-5279) and mouse anti-Hsp47 (Enzo Life Sciences, M16.10A1). Secondary: donkey anti-goat Alexa Fluor™ 488 (Life Technologies, A-11055), donkey anti-rabbit Alexa Fluor™ 488 (Life Technologies, R37118), donkey anti-rabbit Alexa Fluor™ 546 (Life Technologies, A10040) streptavidin Alexa Fluor™ 488 (Life Technologies, S32354) and donkey anti-mouse Cy™5 AffiniPure (Jackson ImmunoResearch, 715-175-150). All secondary antibodies were used at 1:200 dilutions. DAPI was used to visualize nuclei. Rhodamine phalloidin (Invitrogen, R415) was used to visualize F-actin at a 1:200 dilution.

#### Microscopy

##### Fixed Sample Imaging

Either point scanning confocal imaging on an LSM-780 (Zeiss) or Airyscan fast Mode acquisition on an LSM-880 (Zeiss) was performed for immunofluorescence staining prepared samples.

##### Live Imaging

Embryos were mounted in either 10μL KSOMaa drops covered with mineral oil (Sigma, M8140) on 35mm glass-bottomed dishes (MatTek, P35G-1.5-14-C), or in 15-well chambered coverslips (Ibidi, 81506) with 60μL of KSOM if part of a pharmacological inhibition experiment. For long-term imaging (timesteps >5 minutes), imaging was performed on an LSM-780 (Zeiss) with XY sample drift compensation (Rabut and Ellenberg, 2004). Short-term time-lapse imaging (timestep = 10 seconds) was performed on an LSM-880 using Airyscan fast Mode acquisition. Both microscopes are equipped with custom-made temperature and gas-controlled chambers (EMBL) set to 37^°^C and 5% CO_2_ during all experiments and C-Apochromat 40× water objectives (Zeiss).

### Quantification and Statistical Analysis

#### Image Analysis

##### Volume Quantification

Volume quantification for time lapses was calculated from the output of a custom-written automatic membrane segmentation pipeline (Table S2). Volume threshold for segmentation was set to 1pL, 5pL, or 10pL depending on the image quality of the dataset to ensure fidelity.

For vesicles and single timepoints volume was estimated manually by measuring the vesicular or luminal circumference of the central plane in FIJI to extract the radius assuming isotropy.

##### Polarity Phenotype Scoring

Immunofluorescence images were examined for microlumina, apicosome-like structures and lumen-facing membranes expressing pERM. Binary scores were assigned to images on a presence (1), absence (0) basis for each structure, and the frequency of occurrence determined from the binary scores.

##### Microluminal and Cytoplasmic Signal Quantification

A minimal square enclosing FGF4-mNeonGreen positive microluminal membranes was drawn in the central z-plane of the microlumina. A square of equivalent dimensions was drawn in an adjacent cytoplasmic region. The fluorescence intensity was summed in each region for comparison. Background fluorescence (noise; measured in areas of the image not occupied by the embryo) was quantified in the same manner, averaged and subtracted during analysis.

##### Center of Mass Distance to Lumen Surface

The lumen was segmented as described in *Volume Quantification*. The ‘segmented cell mass’ is taken to be the sum of all cells in the embryo such that the total embryo volume is equivalent to the sum of the ‘segmented cell mass’ and the ‘segmented lumen.’ ‘Lumen boundaries’ were determined from the lumen segmentation. ‘Cell mass boundaries’ were determined from the sum of the cell mass and lumen segmentations. Convex hulls representing the lumen and the embryo surface were created from the two boundary point sets. The center of mass of the lumen ($P_{1}$) and the center of mass of the cell mass ($P_{2}$) were used to determine the embryonic-abembryonic axis ($\overset{↔}{L}$). Lumen boundary and embryo outer boundary intersection points with $\overset{↔}{L}$ ($P_{3}$ and $P_{4}$ respectively) were found by recursively searching the facets of the convex hulls such that $\bar{P_{3}P_{4}}$ contains $P_{2}$ but does not contain $P_{1}$. The ICM signal of interest was automatically segmented and the weighted center of mass ($P_{5}$) calculated. $\bar{P_{3}P_{5}}$ is taken as the absolute distance of the signal of interest to the ICM-lumen interface, which is then normalized by the absolute ICM width ($\bar{P_{3}P_{4}}$). This normalization is done to ensure no intrinsic bias due to changes in tissue morphology or size are introduced. The normalized distance for timepoint *t*_*n*_ in a time-lapse starting at t_0_, is ${\left({\bar{d_{t_{n}}}(\bar{w_{t_{n}}})}^{-1}\right)/(\bar{d_{t_{0}}}(\bar{w_{t_{0}}})}^{-1})$ where $\bar{d}=\;\bar{P_{3}P_{5}}$ and $\bar{w}=\;\bar{P_{3}P_{4}}$. All segmentations and calculations were performed automatically using custom Python scripts (Table S2).

As the ICM width along the embryonic-abembryonic axis inherently shrinks throughout blastocyst development, the lack of global movement of all tracked signals toward the lumen confirms ICM width normalization to be a valid measure for positional normalization when examining signals within ICM populations relative to other objects along or relative to the embryonic-abembryonic axis.

##### Fate Specification Analysis

For immunofluorescence images, nuclei were segmented using DAPI signal as a reference. Transcription factor signal was then measured in each nucleus for all channels. From these measurements, a sum measurement was calculated for each channel and subsequently normalized to the reference DMSO control category. The entire analysis was performed using custom Python scripts (Table S2).

##### Spatial Segregation Analysis

Using the nuclear segmentations acquired during *Fate Specification Analysis*, the center of mass was calculated for each nucleus and stored as a 3D coordinate. The median and mean of each dimension from all nuclear centers of mass were calculated resulting in two 3D points of reference within the embryo, such that the median ($P_{1}$) will be within the lumen and the mean ($P_{2}$) will be within the ICM. A 3D line representing the embryonic-abembryonic axis ($\overset{↔}{L}$) was determined from $P_{1}$ and $P_{2}$. A 3D line segment ($\bar{d}$) is drawn from the center of mass of a nucleus ($P_{3}$) to an intersection point with $\overset{↔}{L}$ ($P_{4}$) such that the angle of intersection is 90°. The 3D embryo can then be represented on a 2D coordinate system in which the *x* axis is $\overset{↔}{L}$ such that *x* = 0 is the minimum of all $P_{4}$ points identified and *y* values are the length of $\bar{d}$. After this 3D to 2D projection, the fluorescence intensity values of channels of interest were plotted along a third axis (z) against the positional information (*x,y*). A multidimensional kernel density estimate (KDE) is then performed to acquire a probability density map. KDEs from different channels of interest are then integrated over one another in 3D to obtain a single scalar value representing the degree of sorting between groups (‘Overlap’). Maximum potential overlap values (‘Simulation’) are derived from the ranges of expression and domain sizes observed in WT embryos. The entire analysis was performed using custom Python scripts (Table S2).

##### Image Processing for Figures

Images for Figures 1A and 1B and 3 were processed with a multidimensional median filter (size = 10, scipy.ndimage). Images for Figures 1C and 1F and 2 were acquired using Zeiss Airyscan fast Mode and processed using the corresponding automatic 3D deconvolution. Images for the membrane, Sox2::gfp and Pdgfr⍺^H2B-GFP^ of Figure 3 and Videos S2 and S4 are maximum intensity Z-projections. The segmented lumen images in Figure 3 are sum projections.

#### Cell Counts

Total embryo cell numbers for Figures S2, S6, S10, and S11 were determined from the automatic segmentation of nuclear masks (Table S2) based on DAPI signal. The results from automatic segmentations were validated manually in FIJI for a subset of images in each experimental category to check that the difference between the methods was negligible.

#### Statistical Analysis

All graphs were generated and statistical analysis was performed in Python using the scipy statistics package. Two-sided Fisher exact test was used to compare the frequency of observation between two binary datasets (Figures 2 and S2B). Kruskal-Wallis H-test for independent samples (non-parametric ANOVA) was used to test for statistical significance between populations; ^∗∗∗∗^p<0.0001 ^∗∗∗^p<0.001 ^∗∗^p<0.01 ^∗^p<0.05. Sample sizes and p-values are indicated in text, figures and figure legends. No statistical tests were used to predetermine sample sizes.

### Data and Code Availability

The live-imaging datasets of developing embryos are available upon request. Codes for luminal and tissue segmentation (version 0.0.0) developed during this study are available from the following online repository: https://github.com/allysonryan/phd_notebooks.git.

## Supplemental Information

Document S1. Figures S1–S7 and Tables S1 and S2

Document S2. Article plus Supplemental Information
