# Supplementary material for: Lumen Expansion Facilitates Epiblast-Primitive Endoderm Fate Specification during Mouse Blastocyst Formation
Source: Dev Cell. 2019 Dec 16;51(6):684–697.e4. doi: 10.1016/j.devcel.2019.10.011 (PMC6912163; doi:10.1016/j.devcel.2019.10.011)
Supplement: Document S1. Figures S1–S7 and Tables S1 and S2 [file mmc1.pdf]

Developmental Cell, Volume *51*

Supplemental Information

Lumen Expansion Facilitates Epiblast-Primitive Endoderm Fate Specification during Mouse Blastocyst Formation

Allyson Quinn Ryan, Chii Jou Chan, François Graner, and Takashi Hiiragi

Supplementary Figures

A

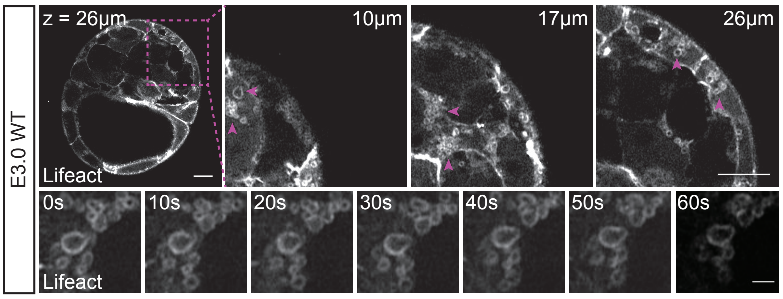

B

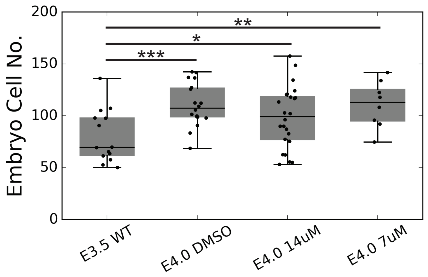

C

n.s.

Exp. / +/-	DMSO	14uM
CC-3 +	1	3
CC-3 -	14	24

Figure S1. Cortically localized, secreted vesicles are not observed after early lumenogenesis (E3.0-E3.5) or in secretion inhibited embryos, which does not impact the total cell number or apoptosis rate of embryos. Related to Figure 1.
(A) Z-slices of an E3.5 WT embryo expressing Lifeact-GFP show only cytoplasmic vesicle clusters (top row, scale bars = 10µm.). Inset (panels 2-4) delineated by magenta box (panel 1). Arrowheads (panels 2-4) highlight vesicle clusters. Time-lapse of vesicle cluster dynamics in an E3.5 WT embryo expressing Lifeact-GFP (bottom row, scale bar = 2µm). Representative of N = 24 embryos. (B) Total embryo cell counts for E3.5-E4.0 COPII inhibition conditions – E3.5 WT, N = 14; E4.0 DMSO, N = 18; E4.0 7µM, N = 8; E4.0 14µM, N = 24. (C) Table showing presence (+) or absence (-) of cleaved caspase-3 signal (CC-3) in DMSO (N = 15) and COPII Inhibition (N = 27) conditions.
*p<0.1 **p<0.01 ***p<0.001
n.s. = not significant

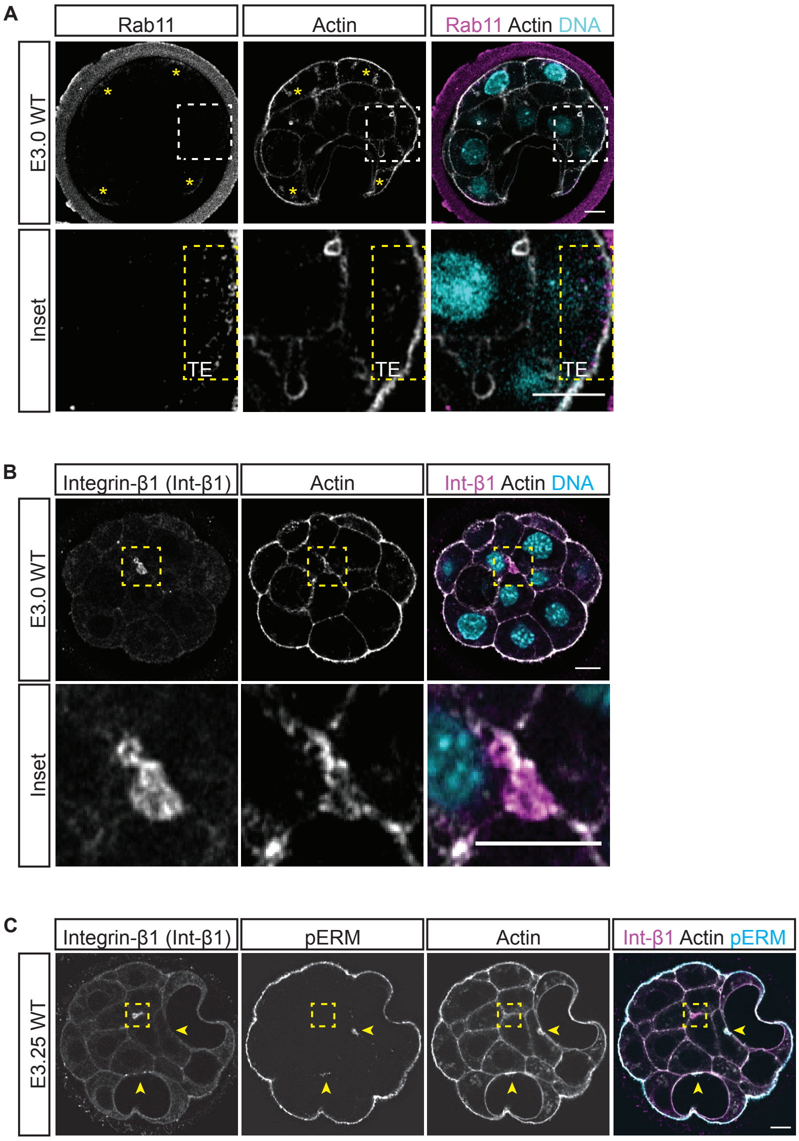

Figure S2. Localization patterns of apico-basally polarized proteins during nascent blastocyst lumen formation. Related to Figure 2.
(A) Immunofluorescence images of Rab11 in an E3.0 WT embryo. Yellow asterisks indicate TE cells in which subapical localization of Rab11 can be seen. Dashed white box outlines inset region. Dashed yellow box outlines apical and subapical region of TE cell in inset. TE cell is labeled as 'TE' within dashed yellow box of inset panels. Representative of N = 14. (B) Immunofluorescence images of Integrin-β1 in an E3.0 WT embryo. Dashed yellow box outlines inset region. Representative of N = 24. (C) Co-immunofluorescence images of Integrin-β1 and pERM in an E3.25 WT embryo. Dashed yellow box highlights Integrin-β1 localized to nascently separated membranes. Yellow arrowheads indicate pERM localized to luminal facing membrane regions.
All scale bars = 10μm
N = number of embryos

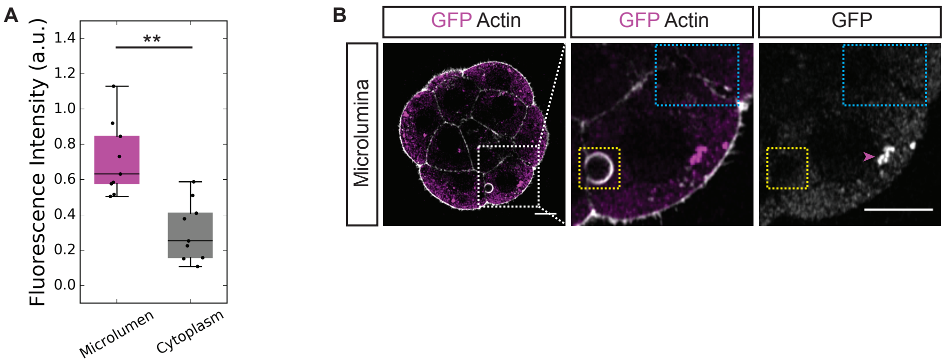

Figure S3. FGF4-mNeonGreen localizes to microlumina, while mNeonGreen does not. Related to Figure 2.

(A) Microluminal and cytoplasmic fluorescence levels within fgf4-mNeonGreen mRNA injected embryos ($N_{Microlumen}$ = 9 microlumina, $N_{Cytoplasm}$ = 9). ** p<0.01 (B) Immunofluorescence images of an E3.0 embryo injected with mNeonGreen mRNA. Blue dashed box outlines region containing multiple microlumina. Yellow dashed box outlines a presumptive fluid contributing vesicle undergoing secretion. Magenta arrowhead indicates a cluster of mNeonGreen protein adjacent to the nucleus. Representative of N = 33 embryos. Scale bars = 10μm.

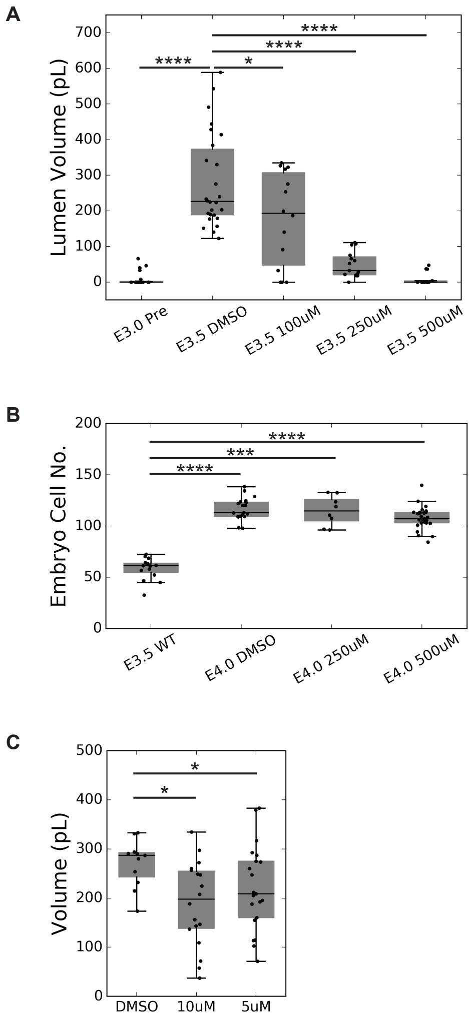

Figure S4. Inhibitory effects of ouabain are dose dependent and do not affect cell number, while CFTR inhibition is less effective. Related to Figure 4.

(A) Boxplot of lumen volume for experimental and control embryos in E3.0-E3.5 ouabain titration inhibitions – E3.0 WT, N = 20; E3.5 DMSO, N = 26; E3.5 100µM, N = 14; E3.5 250µM, N = 15; E3.5 500µM, N = 14. (B) Boxplot of total cell numbers of embryos in experimental and control groups of ouabain titration inhibitions – E3.5 WT, N = 15; E4.0 DMSO, N = 19; E4.0 250µM, N = 8; E4.0 500µM, N = 27. (C) Boxplot of lumen volume in E3.0-E3.5 CFTR inhibition (10µM, N = 18; 5µM, N = 21) and control (DMSO, N = 11) conditions.

*p<0.05 ***p<0.001 ****p<0.0001

N = number of embryos

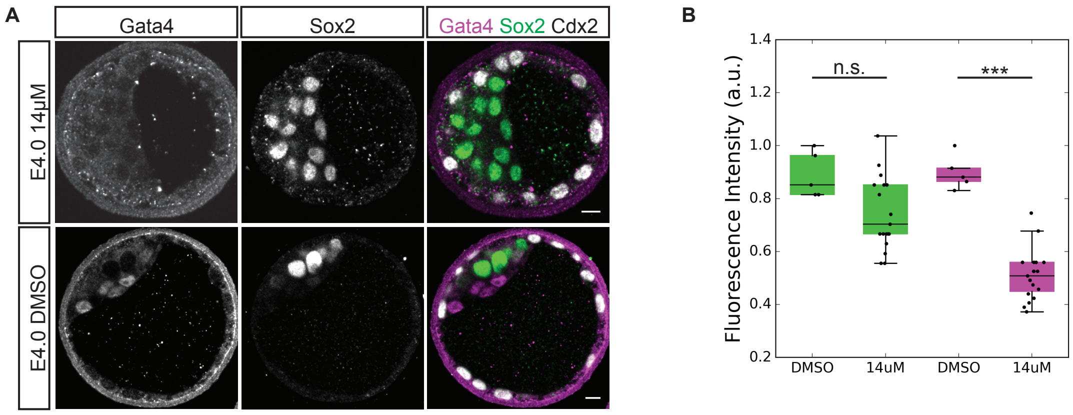

Figure S5. Brefeldin A prevents the formation of the primitive endoderm. Related to Figure 4.
(A) Immunofluorescence images of EPI (Sox2) and PrE (Gata4) fate in COPII inhibited (E4.0 14 μM) and control embryos (E4.0 DMSO). (B) Boxplot of Sox2 (green) and Gata4 (magenta) expression levels in COPII inhibited (14 μM, N = 19) and control (DMSO; N = 5) embryos.
***$p<0.0001$
n.s. = not significant
N = number of embryos

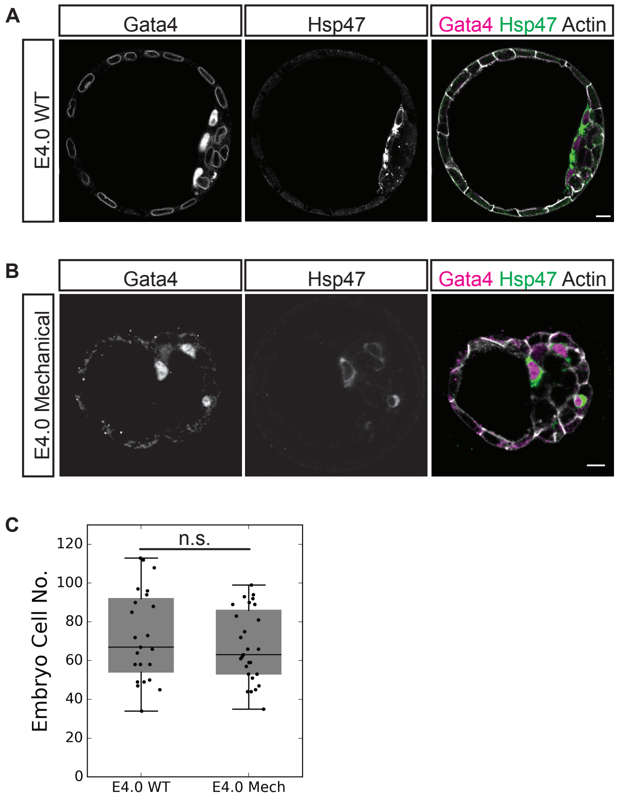

Figure S6. Serial mechanical deflation does not affect tissue specificity of PrE fate markers or embryo cell number. Related to Figure 5.

(A) Immunofluorescence images of Gata4 and Hsp47 in an E4.0 WT embryo. Representative of N = 8. (B) Immunofluorescence images of Gata4 and Hsp47 in an E4.0 mechanically deflated embryo. Representative of N = 9. (C) Boxplot of total cell numbers of embryos in experimental (E4.0 Mech, N = 27) and control (E4.0 WT, N = 23) groups for serial mechanical deflation.

n.s. = not significant.

N = number of embryos

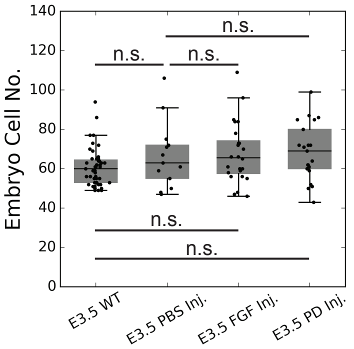

Figure S7. Luminal deposition of PBS, FGF4 protein solution or FGF inhibitor solution does not affect embryo cell number. Related to Figure 6.
Boxplot of total cell numbers of embryos in experimental (E3.5 FGF Inj., N = 24; E3.5 PD Inj.; N = 21) and control (E3.5 WT, N = 47; E3.5 PBS Inj., N = 13) groups for luminal deposition.
n.s. = not significant
N = number of embryos

Supplementary movies

Movie S1. Vesicles localize to all basolateral and apolar membranes prior to visible extracellular fluid accumulation. Related to Figure 1.
Z-stack of actin signal (phalloidin) in a WT E3.0 embryo. Scale bar = 10μm.

Movie S2. Vesicles are actively secreted into intercellular space. Related to Figure 1.
Maximum intensity Z-Projection time-lapse (timestep = 10 seconds) of actin localization (Lifeact-GFP) during vesicle secretion in a WT E3.0 embryo. Scale bar = 5μm.

Movie S3. WT localization of vesicles is maintained in Atp1 inhibited embryos. Related to Figure 1.
Z-stack of actin signal (Lifeact-GFP) in an Atp1 inhibited E3.0 embryo. Scale bar = 10μm.

Movie S4. Vesicles continue to be secreted into intercellular space during Atp1 inhibition. Related to Figure 1.
Maximum intensity Z-projection time-lapse (timestep = 10 seconds) of actin localization (Lifeact-GFP) during vesicle secretion in an Atp1 inhibited E3.0 embryo. Scale bar = 5μm.

Movie S5. Apicosome-like structures are contained in cells isolated from luminal contact. Related to Figure 2.
Z-stack of a cell containing an apicosome-like structure (pERM, magenta; Actin, gray; nuclei, cyan). Scale bar = 5μm.

Movie S6. Apicosome-like structures are released into luminal space when the cell gains a contact-free surface. Related to Figure 2.
Time-lapse (timestep = 15 minutes, hh:mm) of membrane signal (mT) in a cell releasing an apicosome-like structure into luminal space once the cell acquires a contact-free surface along the ICM-lumen interface. Scale bar = 10 μm.

Supplementary tables

Table S1. Genotyping primers. Related to STAR Methods.

Mouse Line	Primer ID	Primer Sequence	PCR Product Size, bp
Lifeact-EGFP	LifeAct for 2	TCAAGAAATTCGAAAGCATCTCAAAGG	TG allele = 725bp
	VenCeru-geno rev	GACCATGTGATCGCGCTTCTCGTT	
mTmG	oIMR7318	CTCTGCTGCCTCCTGGCTTCT	WT allele = 330bp<br>KI allele = 250bp
	oIMR7319	CGAGGCGGATCACAAGCAATA	
	oIMR7320	TCAATGGGCGGGGGTCGTT	
Pdgfra$^{H2B-GFP}$	oIMR7801	CCCTTGTGGTCATGCCAAAC	WT allele = 451bp<br>KI allele = 242bp
	oIMR7802	GCTTTTGCCTCCATTACACTG	
	oIMR7803	ACGAAGTTATTAGGTCCCTCGAC	
Sox2-GFP	GFP-qPCR2-F	CACATGAAGCAGCACGACTT	TG allele = 440bp
	GFP-qPCR3-R	GAACTCCAGCAGGACCATGT	

Table S2. Key Python 2.7 functions and packages used in custom image analysis pipelines. Related to Figures 3-5 and STAR Methods.

Analysis	Package	Key Functions
Lumen Segmentation	scipy.ndimage	median_filter, binary_opening, binary_closing, label
	numpy	percentile, sum
Reporter Expression Center of Mass	scipy.ndimage	median_filter, measurements.center_of_mass
Centroid Distance to Lumen Surface	scipy.spatial	distance.cdist, ConvexHull
	skimage.segmentation	find_boundaries
	numpy	where
	scipy.ndimage	binary_fill_holes
	sympy	geometry.line3d.Line3D, Point3D, intersection
Fate Specification	scipy.ndimage	distance_transform_edt, label
	skimage.feature	peak_local_max
	skimage.morphology	watershed
	numpy	unique, mean, sum
Spatial Segregation	scipy.ndimage	measurements.center_of_mass
	numpy	mean, median
	sympy.geometry	line3d.Line3D, distance, perpendicular_segment, intersection
Luminal Proximity	scipy.ndimage	binary_dilation
Cell Counts	scipy.ndimage	median_filter, binary_opening, measurements.label
